# Quantitative electroencephalography as a marker of cognitive fluctuations in dementia with Lewy bodies and an aid to differential diagnosis

**DOI:** 10.1016/j.clinph.2018.03.013

**Published:** 2018-06

**Authors:** Myrto Stylianou, Nicholas Murphy, Luis R. Peraza, Sara Graziadio, Ruth Cromarty, Alison Killen, John T. O' Brien, Alan J. Thomas, Fiona E.N. LeBeau, John-Paul Taylor

**Affiliations:** a Institute of Neuroscience, Newcastle University Medical School, Newcastle University, Newcastle upon Tyne, UK; b NIHR Newcastle In Vitro Diagnostics Co-operative, Newcastle Upon Tyne Hospitals Foundation Trust, Newcastle upon Tyne, UK; c Department of Psychiatry, University of Cambridge, Cambridgeshire and Peterborough NHS Foundation Trust, Cambridge, UK

**Keywords:** Dementia with Lewy bodies, Alzheimer's disease, Parkinson's disease dementia, Cognitive fluctuations, Quantitative electroencephalography

## Abstract

**Objective:**

We investigated for quantitative EEG (QEEG) differences between Alzheimer’s disease (AD), dementia with Lewy bodies (DLB) and Parkinson’s disease dementia (PDD) patients and healthy controls, and for QEEG signatures of cognitive fluctuations (CFs) in DLB.

**Methods:**

We analysed eyes-closed, resting state EEGs from 18 AD, 17 DLB and 17 PDD patients with mild dementia, and 21 age-matched controls. Measures included spectral power, dominant frequency (DF), frequency prevalence (FP), and temporal DF variability (DFV), within defined EEG frequency bands and cortical regions.

**Results:**

DLB and PDD patients showed a leftward shift in the power spectrum and DF. AD patients showed greater DFV compared to the other groups. In DLB patients only, greater DFV and EEG slowing were correlated with CFs, measured by the clinician assessment of fluctuations (CAF) scale. The diagnostic accuracy of the QEEG measures was 94% (90.4–97.9%), with 92.26% (80.4–100%) sensitivity and 83.3% (73.6–93%) specificity.

**Conclusion:**

Although greater DFV was only shown in the AD group, within the DLB group a positive DFV – CF correlation was found. QEEG measures could classify DLB and AD patients with high sensitivity and specificity.

**Significance:**

The findings add to an expanding literature suggesting that EEG is a viable diagnostic and symptom biomarker in dementia, particularly DLB.

## Highlights

• EEG slowing was evident in dementia with Lewy bodies (DLB) and Parkinson’s disease dementia (PDD) and less in Alzheimer’s disease (AD) patients compared to controls.
• Dominant rhythm variability was larger in AD but only correlated with cognitive fluctuations in DLB.
• QEEG variables classified DLB and AD patients with high sensitivity and specificity.

## 1 Introduction

Dementia with Lewy bodies (DLB) is a common type of dementia after Alzheimer’s disease, accounting for approximately 10–15% of cases at autopsy (McKeith et al., 2004). DLB is associated with quality of life and significant carer burden. It is frequently underdiagnosed and often misdiagnosed as AD, especially at early stages where both diseases manifest with similar cognitive deficits (Metzler-Baddeley, 2007). Estimates of sensitivity and specificity for DLB diagnosis using established clinical criteria (McKeith et al., 2017) have been quite variable but have a common tendency for relatively high specificity but lower sensitivity (Huang et al., 2013). The fact that DLB patients are sensitive to neuroleptics (McKeith et al., 1992), and demonstrate a faster disease progression compared to other dementias (Ballard et al., 2001), underpin the necessity to improve diagnostic accuracy for this group of patients.

Cognitive fluctuations (CFs) are one of the core symptoms of DLB and refer to spontaneous alterations in cognition, attention and arousal (McKeith et al., 2017). CFs are of clinical importance as they have been correlated with visual hallucinations (Varanese et al., 2010), impairment in daily activities and care burden. Moreover, CFs are an important diagnostic feature for DLB as their prevalence reaches 90% of cases, compared to just 20% of AD and 29% of Parkinson’s disease dementia (PDD; Ballard et al., 2002). CFs are also qualitatively different between DLB and AD as in the former case they relate more to executive and perceptual performance, while in the later they are primarily linked to memory impairment (Zupancic et al., 2011). The Clinician Assessment of Fluctuation (CAF) is a clinical scale devised for the psychometric assessment of CFs (Walker et al., 2000). Although CAF is regarded as a fairly reliable measure of CFs if used by an experienced clinician (Van Dyk et al., 2016), the high variability in fluctuation severity and duration of confusional episodes, along with difficulties for informants in separating out what are true intrinsic fluctuations from what are simply responses to external stressors, impose a considerable limitation in CF identification (Bradshaw et al., 2004).

Electroencephalography is an emerging modality for differential diagnosis between dementia subtypes as it is simple, cost-effective, easily accessible and non-invasive compared to imaging approaches. The most prominent QEEG finding in DLB and PDD is a shift of power and dominant frequency (DF) from the alpha frequency range towards high-theta, described as “EEG slowing”. This EEG slowing is most prevalent posteriorly (Briel et al., 1999) and although it is also observed in AD patients (Jackson et al., 2008), it is not as prominent as in the Lewy body diseases – DLB and PDD. In studies quantifying differences between DLB or DLB/PDD, or AD and controls, QEEG variables such as coherence (Snaedal et al., 2012), temporal dominant frequency variability (DFV) (Andersson et al., 2008), power ratio between bands and statistical measures such as Granger causality (Garn et al., 2017), have all achieved high diagnostic sensitivity and specificity, reaching 100% in the latter study.

Previous investigations have found electrophysiological correlations of CFs in DLB patients. Early work using quantitative electroencephalography (QEEG) has shown a correlation between epoch-by-epoch DFV and CFs in DLB patients compared with healthy controls (Walker et al., 2000). Later work also showed that DLB patients with CFs had greater DFV compared to AD patients in posterior brain regions, and used the DFV together with other QEEG measures to classify AD, PDD-CFs, PDD-without CFs and DLB patients and controls (Bonanni et al., 2008). More recently, a multi-centre cohort analysis has verified these results (Bonanni et al., 2016).

The aforementioned findings of QEEG signatures in DLB in addition to the fact that the QEEG measures were shown to be correlated with the clinical phenotype of DLB and specifically with CFs, suggest that the QEEG could be utilised to investigate for a neurophysiological divergence between DLB and other dementias. The QEEG investigations performed so far have not yet managed to identify differences (Engedal et al., 2015, Garn et al., 2017) between DLB and PDD. Generally, these Lewy body dementia (LBD) subtypes demonstrate great similarities in neuropathological processes, symptom manifestation and treatment. However, DLB is typically characterised by greater executive dysfunction, more psychiatric symptoms, poorer response to levodopa (L-DOPA) and greater amyloid burden compared to PDD (Edison et al., 2008). Moreover, the onset of motor symptoms precedes that of dementia in PDD while in DLB, dementia appears concurrently or before motor symptoms (McKeith et al., 1992). These discrepancies may indicate differences in the spatio-temporal sequence of pathology, with a predominant brain-stem start and rostral progression in PDD and a cortical inception in DLB (Beyer et al., 2007). Potential QEEG differences between PDD and DLB are of research interest, as they could provide insight for better understanding these LBD subtypes.

Earlier QEEG studies focused on investigating the capacity of such measures in aiding DLB differential diagnosis in clinical settings. Hence, they utilized methods such as assessment by visual observation (Bonanni et al., 2008), or attempted to develop an online method that performs analysis during and right-after EEG acquisition (Garn et al., 2017). Here we took a less clinically-orientated approach, as our primary goal was to characterise and compare the resting EEG rhythm in AD, DLB and PDD patients and in relation to healthy controls, and to investigate for DLB specific signatures of CFs. Thus, we performed extensive pre-processing analysis of the EEG signal and a thorough analysis for differences in QEEG measures within different frequency ranges and brain regions, between diagnostic groups. Based on the literature, we hypothesized that dementia patients will exhibit a differential pattern in the distribution of QEEG measures of power and DF within different frequency ranges compared to healthy controls, and that these QEEG measures in addition to DF variability in time (DFV) will also differ between the dementia groups. We also hypothesized that greater DFV will only characterise LBDs and possibly only DLB, and that greater DFV will correlate with more CFs within these groups. Finally, to assess the possible utility of these measures in the development of biomarkers, the QEEG measures that were found to be significantly different between groups were used to predict dementia diagnosis.

## 2 Material and methods

### 2.1 Diagnostic groups

Initially we pre-processed EEG data from 21 healthy controls, 19 AD, 20 DLB and 20 PDD participants (Table 1 for the demographic data of the final groups). Patients were individuals who were referred to local old age psychiatry and neurology services and diagnosis was determined by two independent experienced clinicians (Alan J. Thomas and John-Paul Taylor). Controls were age-matched volunteers. Patients with DLB fulfilled the 2005 and 2017 revised criteria for probable DLB (McKeith et al., 2005, McKeith et al., 2017) and patients with PDD fulfilled the criteria for probable PDD (Emre et al., 2007). Individuals with AD met the revised criteria of the National Institute of Neurological and Communicative Diseases and Stroke/AD and Related Disorders Association for probable AD (McKhann et al., 2011). The CAF score was assessed by the clinicians and CFs were defined on the basis that they were typical of those seen in DLB and internally driven rather than a response to external environmental factors. Healthy participants demonstrated no evidence of dementia as determined by the Cambridge Cognitive Examination (CAMCOG) score (>80) and from clinical history. Exclusion criteria for all participants included significant history of neurological or psychiatric conditions. Prescriptions of acetylcholinesterase inhibitors (AChEIs), memantine and dopaminergic medications were allowed. Ethical approval was provided by the Northumberland Tyne and Wear NHS Trust and Newcastle University ethics committee.

**Table 1:** Demographics table for the healthy control (N = 21), Alzheimer’s disease (AD; N = 18), dementia with Lewy bodies (DLB; N = 17) and Parkinson’s disease dementia (PDD; N = 17) groups that were used for our analysis. L-DOPA = levo-dopa, LED = L-DOPA equivalent dose, AChEIs = acetylcholinesterase inhibitors, MMSE = Mini mental state examination, CAF = Clinician’s assessment of fluctuations scale, UPDRS = Unified Parkinson’s disease rating scale, NPI = Neuropsychiatric inventory total score. Although it is not shown in the table, 1 PDD patient (5.9%) was on memantine.

	Controls (N = 21)	AD (N = 18)	DLB (N = 17)	PDD (N = 17)
Age in yrs ± SD	76.19 ± 5.32	76.06 ± 7.81	75.71 ± 5.34	75.44 ± 4.66
Males (%)	66.7%	88.9%	88.2%	100%
L-DOPA	–	0%	52.9%	100%
LED	–	0%	348.94	423.42
AChEIs	–	94.4%	88.2%	76.5%
Age at diagnosis (yrs ± SD)	–	74.64 ± 7.63	73 ± 5.11	74.07 ± 6.29
Diagnosis duration (yrs ± SD)	–	1.5 ± 0.9	1.08 ± 0.70	0.94 ± 0.73
MMSE	29.19 ± 0.87	23.67 ± 1.68	25 ± 2.89	23.94 ± 2.59
CAF	–	0.47 ± 0.87	2.76 ± 3.78	6.59 ± 4.29
NPI total	–	7.29 ± 7.61	8 ± 5.27	20.35 ± 12.9
UPDRS	1.14 ± 1.42	1.67 ± 1.61	13.82 ± 5.32	27.06 ± 11.44

### 2.2 EEG recordings

High-density, eyes-closed resting-state EEG recordings were obtained using 128 channel ANT Waveguard caps (ANT Neuro, Netherlands) with an Ag/AgCl electrode montage set according to the 10-5 placement system (Oostenveld and Praamstra, 2001). Electrode impedance with kept below 5 kΩ. A reference electrode (Fz) was used, no filters were applied during acquisition and the sampling frequency was set at 1024 Hz. The patients that received medication had normally taken AChEIs at least 4 h before while the time of the last Levodopa dose was 1–3 h prior to the EEG session.

### 2.3 Pre-processing

Pre-processing of the EEG recordings was performed off-line after acquisition on the MATLAB environment (MATLAB 8.5, The MathWorks Inc., Natick, MA, 2015), using the EEGLAB toolbox version 13 (Delorme and Makeig, 2004). The EEG signal was filtered with a 4 Hz high-pass and a 46 Hz low-pass filter. Lower frequencies were filtered out as they imposed noise on the higher frequencies that were of more interest, and because the EEG generally has a limited accuracy in estimating very low and very high frequencies (Niedermeyer and Lopes da Silva, 2004). A notch filter was applied at 50 Hz. Recordings from all electrodes were visually inspected in the power-time domain and rejected if they had a kurtosis value over 3, or if they contained clear and consistent artifacts such as electrooculogram (EOG) and electromyogram (EMG) artifacts. The number of channels removed was kept to the minimum possible (mean = 17.7 ± 6.7, min. = 0, max. = 33).

Independent component analysis (ICA) was used to accurately estimate and remove the presence of additional ocular, muscular, and other neuronal activity (Kropotov and Kropotov, 2009). Individual recordings were reduced to 30 principal components and then decomposed using the extended RUNICA algorithm (Bell and Sejnowski, 1995, Delorme and Makeig, 2004). Components representing existing templates for muscular, ocular, and electrical (50 Hz line noise) artefacts (Jung et al., 2000) were rejected (mean = 5.2 ± 1.6, min. = 0, max. = 9) and the remaining ICs remixed. The recordings were then segmented into 2-s long epochs and were inspected for any remaining artefacts. Epochs containing large artifacts were removed across channels, in a conservative manner. Finally, the removed channels were replaced using spherical spline interpolation (Ferree, 2006). As a final step, the EEG montage was changed to average reference.

### 2.4 Variable extraction

**Fig. 1:**
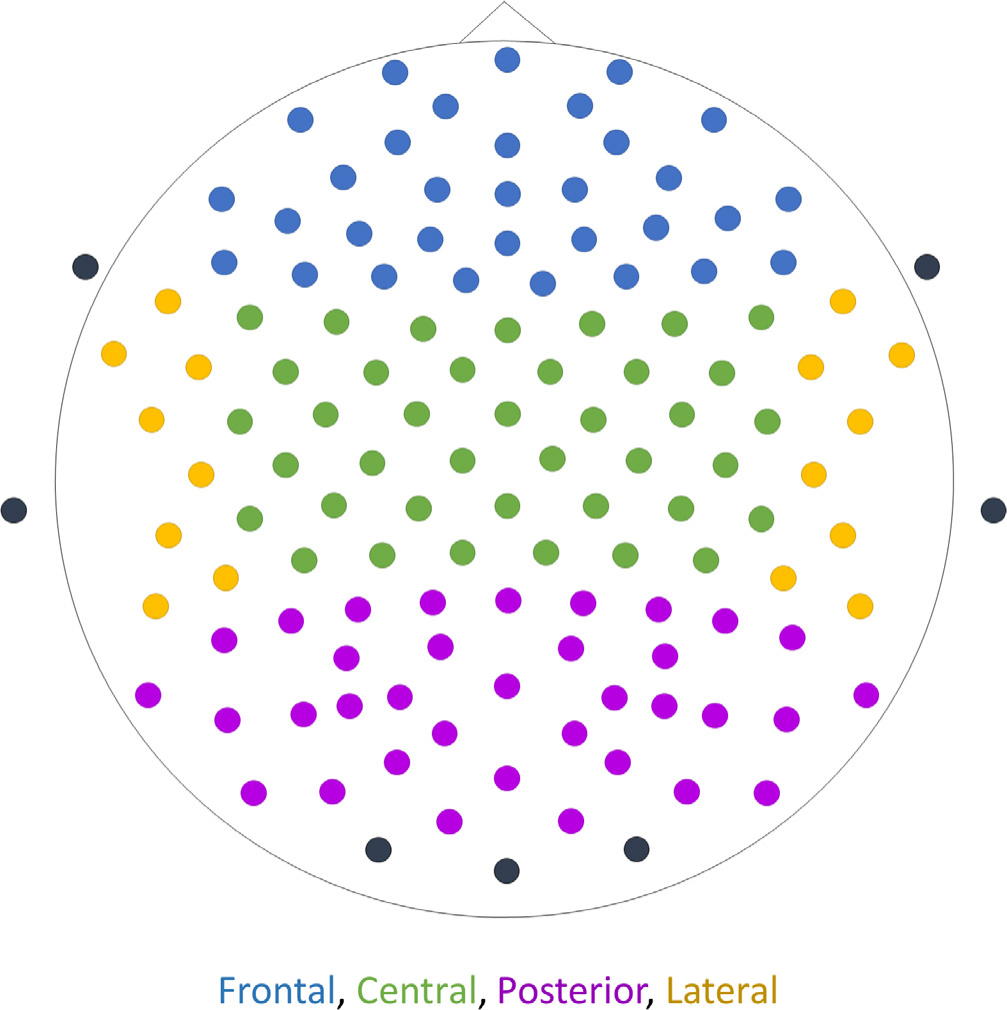
Placement of the 128 electrodes according to the 10-5 placement system. The signal recorded from the electrodes indicated with black colour was selected out as it was deemed too noisy. The colours indicate the grouping of the electrodes into four regions: blue = frontal, green = central, purple = posterior, yellow = lateral. (For interpretation of the references to colour in this figure legend, the reader is referred to the web version of this article.)

The power spectral density (PSD) for each 2-s epoch was estimated using Bartlett’s method (Bartlett, 1950) with a 0.25 Hz frequency resolution using a 4-s FFT (fast Fourier transform) size and a Hamming window, for each electrode. To compensate for the between subject variability in factors such as brain neurophysiology, anatomy and physical tissue properties, the data were transformed to relative power spectral density (rPSD; Eq. (1); Rodriguez et al., 1999). The rPSD was extracted for each time point of each epoch (sampling frequency = 1024 Hz), and for each electrode. Then, for each epoch of a recording, the power was averaged across electrodes for each of four regions: frontal, central, posterior and lateral (Fig. 1). Seven subjects were rejected from further analysis due to an insufficient number of clean data (<47 epochs). For the remaining 73 subjects (21 healthy controls, 18 AD, 17 DLB and 17 PDD; Table 1), only the first 47 epochs of extracted power per region were utilised (total length of 94 s).

(1) $$\overset{¯}{g}(f)=\frac{g(f)}{\sum_{f}g(f)}$$

Equation (1): Calculation of the relative PSD/power $\left(\overset{¯}{g}\right)$ across the power spectrum (4–46 Hz). At each point in the frequency spectrum the amplitude (g(f)) is divided by the sum of all amplitudes across the frequency spectrum ($\sum_{f}g(f)$) (Kropotov and Kropotov, 2009).

**Fig. 2:**
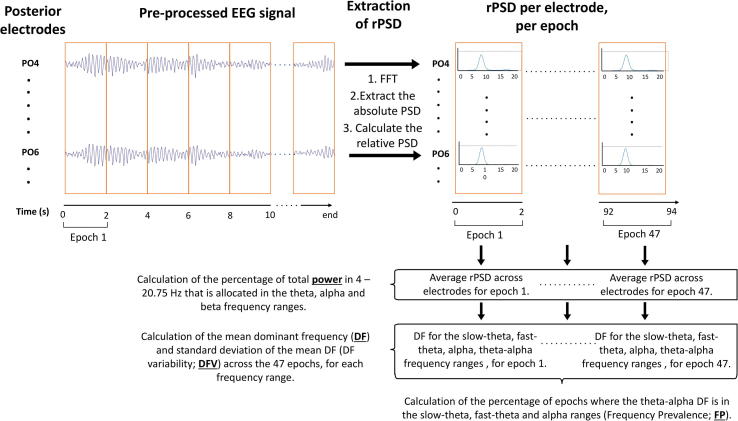
Schematic diagram illustrating the process of extracting each of the four main quantitative EEG variables used in this study, for one participant in the posterior region. The filtered, pre-processed EEG signal on each of the electrodes in posterior derivations (N = 35) is windowed in 2 s long epochs. The signal undergoes fast-Fourier transform (FFT) and using Bartlett’s method the absolute power spectral density (PSD) is calculated for each epoch, for each electrode. The relative PSD (rPSD) is then calculated to normalize the signal. The mean rPSD is obtained across posterior electrodes, for each epoch (up to 47 epochs) of the recording, and the percentage of the total power in the 3–20.75 Hz range allocated to the theta (4–7.75 Hz), alpha (8–13.75 Hz) and beta (14–20.75 Hz) frequency ranges is calculated. The frequency with the highest power within the slow-theta (4–5.5 Hz), fast-theta (5.5–7.75 Hz), alpha and theta-alpha (4–13.75 Hz) frequency ranges was identified within each epoch, and that value corresponded to the dominant frequency (DF). The mean DF and the standard deviation of the mean DF (DF variability; DFV) across epochs were then calculated. Finally, the DF within each epoch was assessed and was characterised to be in the slow-theta, fast-theta or alpha range. The epochs that were characterised by a DF within each of these ranges are shown as a percentage of the total number of epochs. These percentages were the slow-theta, fast-theta and alpha frequency prevalence (FP). The same procedure was followed for the other three cortical regions.

The mean power distributed in each of three frequency bands: theta (4–7.75 Hz), alpha (8–13.75 Hz), beta (14–20.75 Hz), was extracted as a percentage of the total power in that range, across epochs per region (Table 2; Fig. 2). Higher frequencies were excluded as they are prone to contamination by electromyogram rhythms (Whitham et al., 2007). The DF – the frequency with the highest power between 4 Hz and 20.75 Hz – was extracted for each epoch to calculate the mean DF and DF variability (DFV; SD from the mean DF) across epochs, for the slow-theta (4–5.5 Hz), fast-theta (5.5–7.75 Hz; defined by others as pre-alpha; Bonanni et al., 2008), theta, alpha and theta/alpha (4–13.75 Hz) frequency ranges (Table 3; Fig. 2). Since the DF was limited within the theta-alpha range, beta band activity was excluded. The theta-alpha DF was used to calculate the Frequency Prevalence (FP) distribution, which is the percentage of epochs having a DF falling within the slow-theta, fast-theta and alpha frequency ranges (Table 3; Fig. 2). These measures were calculated for each patient, for each diagnostic group and for each band and region combination.

**Table 2:** The mean percentage of the total power distributed in each of three frequency bands: theta (4–7.75 Hz), alpha (8–13.5 Hz), beta (14–30.75 Hz), in each region: frontal, central, posterior, lateral, for each group: healthy controls (N = 21), Alzheimer’s disease (AD; N = 18), dementia with Lewy bodies (DLB; N = 17) and Parkinson’s disease dementia (PDD; N = 17) patients.

	Regions	Controls	AD	DLB	PDD
Theta	Frontal	20.19 ± 5.22	24.57 ± 6.26	37.63 ± 6.36	35.89 ± 7.46
	Central	19.18 ± 4.96	23.02 ± 6.13	36.90 ± 7.05	35.33 ± 6.37
	Posterior	19.79 ± 5.77	23.51 ± 6.63	39.62 ± 7.53	39.10 ± 7.63
	Lateral	19.32 ± 5.29	24.39 ± 6.53	36.96 ± 6.07	35.35 ± 7.30

Alpha	Frontal	35.12 ± 5.66	32.11 ± 4.31	28.96 ± 5.10	27.42 ± 2.57
	Central	34.74 ± 5.11	32.61 ± 4.47	29.84 ± 4.91	29.04 ± 2.28
	Posterior	38.91 ± 6.04	35.49 ± 5.81	29.26 ± 5.75	23.91 ± 2.50
	Lateral	35.41 ± 5.10	33.43 ± 4.00	28.57 ± 4.98	26.75 ± 2.34

Beta	Frontal	44.69 ± 7.09	43.32 ± 7.03	33.40 ± 3.74	36.69 ± 7.52
	Central	46.07 ± 6.75	44.37 ± 6.71	33.26 ± 3.85	35.65 ± 6.20
	Posterior	41.30 ± 7.09	40.99 ± 8.38	31.11 ± 5.36	31.72 ± 7.67
	Lateral	45.27 ± 7.71	42.17 ± 6.59	34.47 ± 5.52	37.90 ± 7.80

**Table 3:** The mean dominant frequency (DF) ± DFV (mean SD of the DF), DFV ± SD and frequency prevalence (FP) ± SD for the theta (4–7.75 Hz), slow-theta (4–5.5 Hz), fast-theta (5.75–7 Hz), alpha (8–13.75 Hz) and theta-alpha (4–13.75 Hz) frequency ranges in each region: frontal, central, posterior, lateral, for each group: healthy controls (N = 21), Alzheimer’s disease (AD; N = 18), dementia with Lewy bodies (DLB; N = 17) and Parkinson’s disease dementia (PDD; N = 17) patients.

	Regions	Variables	Controls	AD	DLB	PDD
Theta	Frontal	DF ± SD	6.93 ± 0.4	6.57 ± 0.54	6.49 ± 0.60	6.26 ± 0.31
		DFV ± SD	0.93 ± 0.25	0.98 ± 0.24	0.73 ± 0.24	0.80 ± 0.19
		FP ± SD	19.55 ± 22.14	44.92 ± 24.54	82.48 ± 21.00	91.36 ± 8.29
	Central	DF ± SD	7.11 ± 0.33	6.67 ± 0.59	6.65 ± 0.52	6.51 ± 0.35
		DFV ± SD	0.81 ± 0.28	0.86 ± 0.29	0.63 ± 0.22	0.67 ± 0.16
		FP ± SD	18.84 ± 24.96	41.02 ± 26.07	80.60 ± 21.02	90.49 ± 9.22
	Posterior	DF ± SD	7.17 ± 0.35	6.71 ± 0.60	6.57 ± 0.66	6.31 ± 0.29
		DFV ± SD	0.78 ± 0.33	0.86 ± 0.31	0.60 ± 0.21	0.72 ± 0.18
		FP ± SD	16.72 ± 23.49	34.99 ± 24.45	83.35 ± 23.68	94.99 ± 6.25
	Lateral	DF ± SD	7.18 ± 0.31	6.80 ± 0.54	6.65 ± 0.53	6.51 ± 0.36
		DFV ± SD	0.80 ± 0.30	0.83 ± 0.33	0.64 ± 0.26	0.72 ± 0.18
		FP ± SD	17.53 ± 22.06	39.24 ± 26.68	83.35 ± 18.76	89.36 ± 15.10

Slow-theta	Frontal	DF ± SD	4.88 ± 0.15	4.93 ± 0.17	5.01 ± 0.15	5.02 ± 0.14
		DFV ± SD	0.55 ± 0.06	0.52 ± 0.07	0.49 ± 0.08	0.50 ± 0.08
		FP ± SD	2.23 ± 4.28	10.52 ± 13.29	16.27 ± 21.06	18.77 ± 11.56
	Central	DF ± SD	4.95 ± 0.17	4.98 ± 0.18	5.16 ± 0.14	5.14 ± 0.13
		DFV ± SD	0.52 ± 0.06	0.49 ± 0.06	0.42 ± 0.10	0.43 ± 0.10
		FP ± SD	1.21 ± 3.05	9.46 ± 12.37	9.51 ± 14	11.14 ± 12.28
	Posterior	DF ± SD	4.91 ± 0.14	4.94 ± 0.18	5.11 ± 0.13	5.11 ± 0.15
		DFV ± SD	0.54 ± 0.06	0.51 ± 0.06	0.45 ± 0.07	0.45 ± 0.10
		FP ± SD	1.11 ± 3.34	6.97 ± 9.25	13.77 ± 23.03	16.15 ± 11.75
	Lateral	DF ± SD	4.86 ± 0.18	4.94 ± 0.16	5.09 ± 0.15	5.09 ± 0.16
		DFV ± SD	0.56 ± 0.05	0.51 ± 0.08	0.44 ± 0.11	0.46 ± 0.09
		FP ± SD	0.51 ± 1.15	5.44 ± 6.74	10.76 ± 17.41	11.76 ± 11.99

Fast Theta	Frontal	DF ± SD	7.20 ± 0.27	6.98 ± 0.32	6.74 ± 0.41	6.55 ± 0.22
		DFV ± SD	0.59 ± 0.14	0.62 ± 0.11	0.53 ± 0.12	0.55 ± 0.09
		FP ± SD	17.32 ± 21.02	34.4 ± 17.95	66.21 ± 20.86	72.59 ± 10.45
	Central	DF ± SD	7.31 ± 0.22	7.02 ± 0.31	6.79 ± 0.43	6.69 ± 0.24
		DFV ± SD	0.53 ± 0.16	0.60 ± 0.14	0.50 ± 0.11	0.52 ± 0.07
		FP ± SD	17.62 ± 24.30	31.56 ± 20.68	71.09 ± 20.34	79.35 ± 11.86
	Posterior	DF ± SD	7.35 ± 0.21	7.06 ± 0.36	6.76 ± 0.47	6.54 ± 0.20
		DFV ± SD	0.52 ± 0.17	0.58 ± 0.16	0.48 ± 0.11	0.52 ± 0.09
		FP ± SD	15.60 ± 22.51	28.01 ± 19.35	69.59 ± 27.37	78.85 ± 11.86
	Lateral	DF ± SD	7.36 ± 0.19	7.09 ± 0.30	6.81 ± 0.41	6.70 ± 0.24
		DFV ± SD	0.50 ± 0.15	0.58 ± 0.18	0.49 ± 0.13	0.52 ± 0.08
		FP ± SD	17.02 ± 21.95	33.81 ± 24.26	72.59 ± 21.64	77.60 ± 15.63

Alpha	Frontal	DF ± SD	9.13 ± 0.57	9.40 ± 0.88	8.64 ± 0.21	9.01 ± 0.44
		DFV ± SD	0.88 ± 0.40	1.07 ± 0.43	0.85 ± 0.32	1.19 ± 0.27
		FP ± SD	80.45 ± 22.14	55.08 ± 24.54	17.52 ± 21	8.64 ± 8.29
	Central	DF ± SD	9.13 ± 0.65	9.42 ± 0.90	8.48 ± 0.17	8.70 ± 0.39
		DFV ± SD	0.82 ± 0.41	1.04 ± 0.40	0.60 ± 0.17	0.87 ± 0.37
		FP ± SD	81.16 ± 24.96	58.98 ± 26.07	19.40 ± 21.02	9.51 ± 9.22
	Posterior	DF ± SD	9.03 ± 0.63	1.06 ± 0.96	8.49 ± 0.18	8.72 ± 0.33
		DFV ± SD	0.64 ± 0.35	0.86 ± 0.45	0.62 ± 0.25	0.93 ± 0.37
		FP ± SD	83.28 ± 23.49	65.01 ± 24.45	16.65 ± 23.68	5.01 ± 6.25
	Lateral	DF ± SD	9.12 ± 0.71	0.92 ± 0.82	8.48 ± 0.18	8.61 ± 0.41
		DFV ± SD	0.82 ± 0.37	0.82 ± 0.40	0.65 ± 0.24	0.84 ± 0.35
		FP ± SD	82.47 ± 22.06	60.76 ± 26.68	16.65 ± 18.76	10.64 ± 15.10

Theta-alpha	Frontal	DF ± SD	8.79 ± 0.75	8.24 ± 1.29	6.75 ± 0.80	6.45 ± 0.63
		DFV ± SD	1.07 ± 0.46	1.29 ± 0.59	0.91 ± 0.27	0.98 ± 0.33
	Central	DF ± SD	8.81 ± 0.82	8.36 ± 1.30	6.93 ± 0.71	6.68 ± 0.76
		DFV ± SD	0.92 ± 0.47	1.30 ± 0.60	0.80 ± 0.25	0.82 ± 0.25
	Posterior	DF ± SD	8.82 ± 0.79	8.65 ± 1.21	6.78 ± 0.86	6.41 ± 0.58
		DFV ± SD	0.78 ± 0.38	1.21 ± 0.75	0.73 ± 0.23	0.84 ± 0.27
	Lateral	DF ± SD	8.88 ± 0.81	8.42 ± 1.13	6.90 ± 0.71	6.74 ± 0.81
		DFV ± SD	0.93 ± 0.41	1.13 ± 0.51	0.79 ± 0.31	0.88 ± 0.35

### 2.5 Statistical analysis

The mean power, theta-alpha DF and theta, alpha and theta-alpha DFV were statistically compared using repeated measures ANOVA, for region as the within-subjects factor and diagnosis as the between-subjects factor. When a significant interaction was found we followed up by univariate ANOVA and post hoc analysis with a Bonferonni correction. The DFV (for all frequency ranges) and the theta-alpha DF values were logarithmically transformed to achieve homogeneity of variance/homoscedasticity. Heteroscedasticity could not be solved for the theta and alpha DF and hence we performed Welch’s ANOVA followed by the Games-Howell test. To statistically compare the distribution of the FP in the slow-theta, fast-theta and alpha frequency ranges we performed Kruskal-Wallis H test followed by post hoc analysis. Pearson’s product-moment correlation and Spearman’s rank correlation were used to investigate for correlations between these variables and the CAF score, the MMSE score and the levodopa equivalent dose (LED), for each diagnostic group. Manual correction for multiple comparisons by appropriating the level of α significance (α/N) was performed for the non-parametric statistical analyses and for the correlation analyses, where Bonferonni correction was not available with the statistical software.

In order to assess the capacity of the QEEG variables that were significantly different between the AD and DLB, and the DLB and PDD groups to predict diagnosis, the generalised estimating equations (GEE) procedure were used. This method allows the analysis of repeated measurements without the assumption for normal distribution (Carr and Chi, 1992). The QEEG variables that introduce multicollinearity to the model (variance inflation factor >5) were excluded from this analysis. Region was defined as the within-subjects variable, diagnosis as the between-subjects variable and the QEEG variables and the CAF score as the co-factors. The variables that significantly predicted diagnosis were then used to calculate the receiver operating characteristic (ROC) curve, and obtain the area under the curve (AUC), sensitivity and specificity with asymptotic confidence intervals. The sensitivity/specificity cut-off was determined using Youden's index.

## 3 Results

### 3.1 Data and demographics

Data from a total of 73 individuals (21 healthy controls, 18 AD, 17 DLB, 17 PDD; Table 1) were further analysed after data extraction. Participants were well matched for age at diagnosis and age at the time of the recording (p > 0.05), as well as MMSE score (p > 0.05). The PDD and DLB groups had significantly higher CAF scores than AD patients (p < 0.01; p < 0.05 respectively), with the PDD group also having a higher CAF score than the DLB group (p < 0.01). Lastly, the neuropsychiatric inventory (NPI) total and Unified Parkinson’s disease rating scale (UPDRS) scores were higher in the DLB/PDD subjects compared to the other groups, and in the PDD compared to the DLB group (p < 0.01).

### 3.2 EEG slowing

**Fig. 3:**
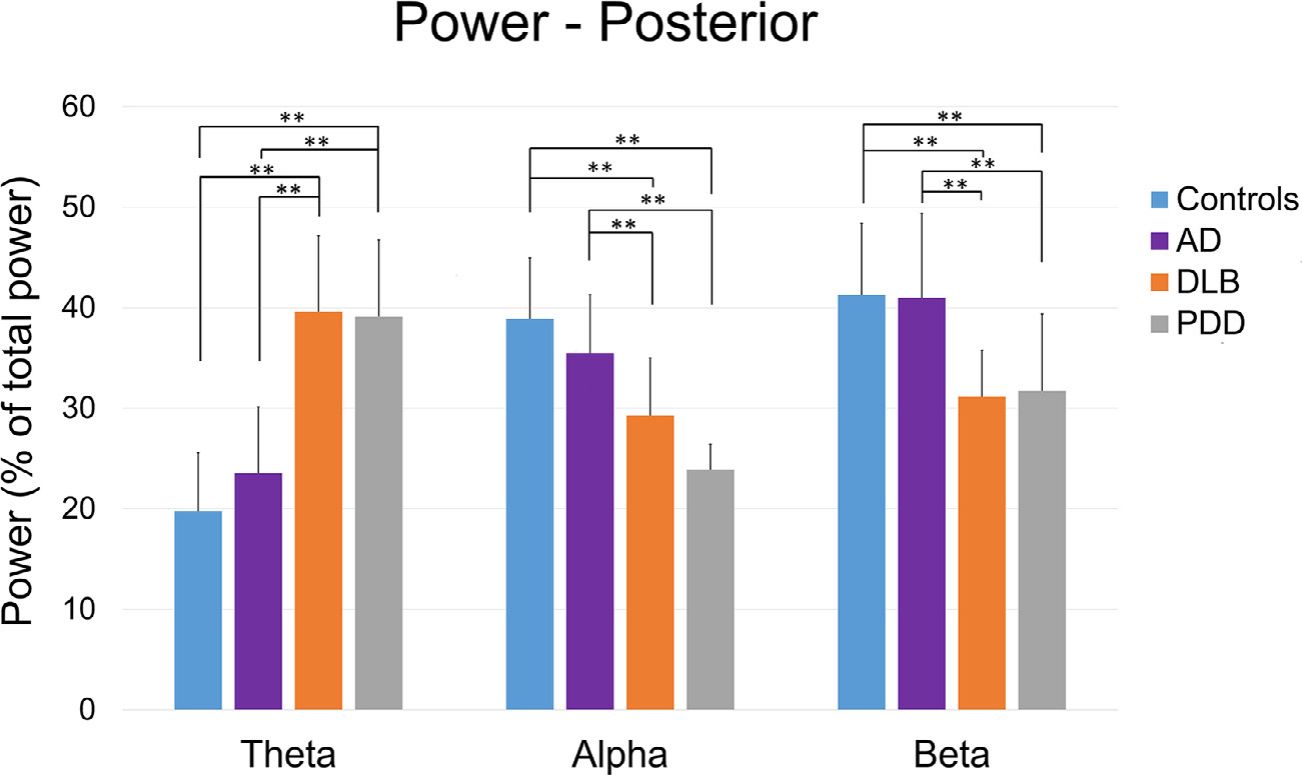
The mean percentage distribution of the total relative power in three frequency bands (Hz): theta (4–7.75), alpha (8–13.5) and beta 20.75), for each of four diagnostic groups: healthy controls (N = 21), Alzheimer’s disease (AD; N = 18), dementia with Lewy bodies (DLB; N = 17) and Parkinson’s disease dementia (PDD; N = 17) patients, for the posterior region. Similar observations were made in the frontal, central and lateral regions but are not shown. Error bards indicate the standard deviation.

We found a significant effect of diagnosis on the mean power in the theta: *F (3, 69) = 39.48, p < 0.01,* alpha: *F (3, 69) = 14.49, p < 0.01* and beta: *F (3, 69) = 12.825, p < 0.01* ranges (Table 2; Fig. 3). In all regions, PDD and DLB groups had higher theta power than AD patients and healthy controls (*p < 0.01*). In the alpha and beta ranges the opposite pattern was observed. Specifically, in the alpha band, controls had significantly higher power than PDD patients in all regions (*p < 0.01*), and compared to DLB patients frontally, posteriorly and laterally (*p < 0.01*). Moreover, AD patients had greater alpha power than PDD patients posteriorly and laterally (*p < 0.01*), and also to DLB patients frontally (*p < 0.05*), posteriorly and laterally (*p < 0.01*). In the beta range, DLB patients had lower power than AD patients and controls in all regions (*p < 0.01*). PDD patients had lower power than healthy controls frontally and centrally (p < 0.01) and posteriorly and laterally (p < 0.05), and from AD patients frontally, posteriorly (*p < 0.05*) and centrally (*p < 0.01*).

**Fig. 4:**
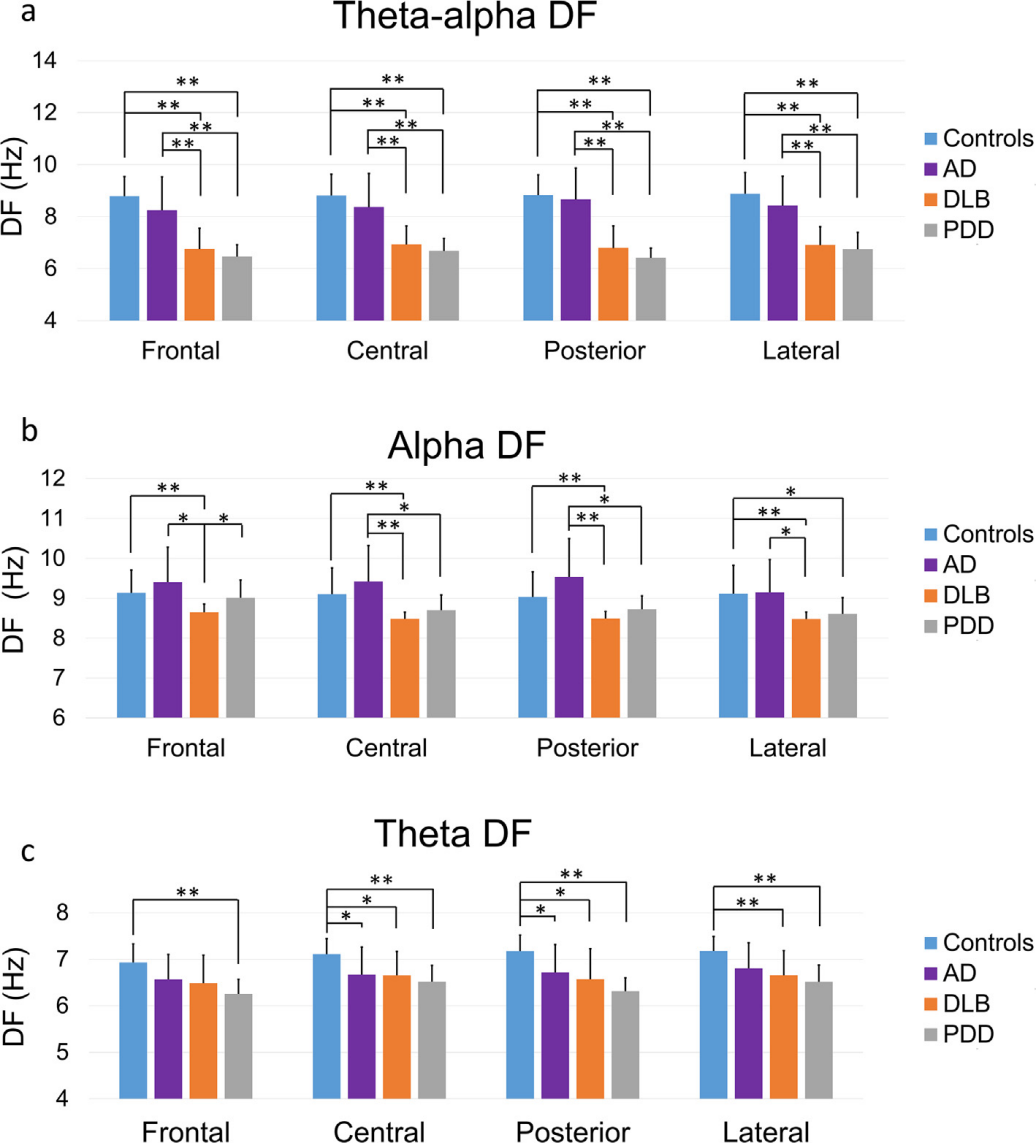
The mean dominant frequency (DF) in the theta-alpha (4–13.75 Hz), alpha (8–13.75 Hz) and theta (4–7.75 Hz) frequency ranges, for each of four diagnostic groups: healthy controls (N = 21), Alzheimer’s disease (AD; N = 18), dementia with Lewy bodies (DLB; N = 17) and Parkinson’s disease dementia (PDD; N = 17) patients, in the frontal, central, posterior and lateral regions. Error bars indicate the standard deviation (SD), ^**^p < 0.05, ^**^p < 0.01.

We also found a significant effect of diagnosis in the second measure of interest, the mean theta-alpha DF (*F (3, 69) = 36.78, p < 0.01),* which was significantly higher in all cortical regions in controls and AD patients compared to the other patient groups (Table 3, Fig. 4). The mean theta DF was significantly higher in controls compared to the PDD group frontally, to the AD, DLB and PDD groups centrally and posteriorly*,* and to the DLB and PDD groups laterally. Significant differences were also found between groups in the alpha DF, in all regions. Specifically, the DLB group had significantly lower alpha DF than the control and AD group in all regions. The PPD group had higher alpha DF than the DLB group frontally, the AD group centrally and posteriorly and the control group laterally (Fig. 4). A trend for a greater alpha DF in the AD compared to the control group was observed, but was not verified by the statistical analysis.

**Fig. 5:**
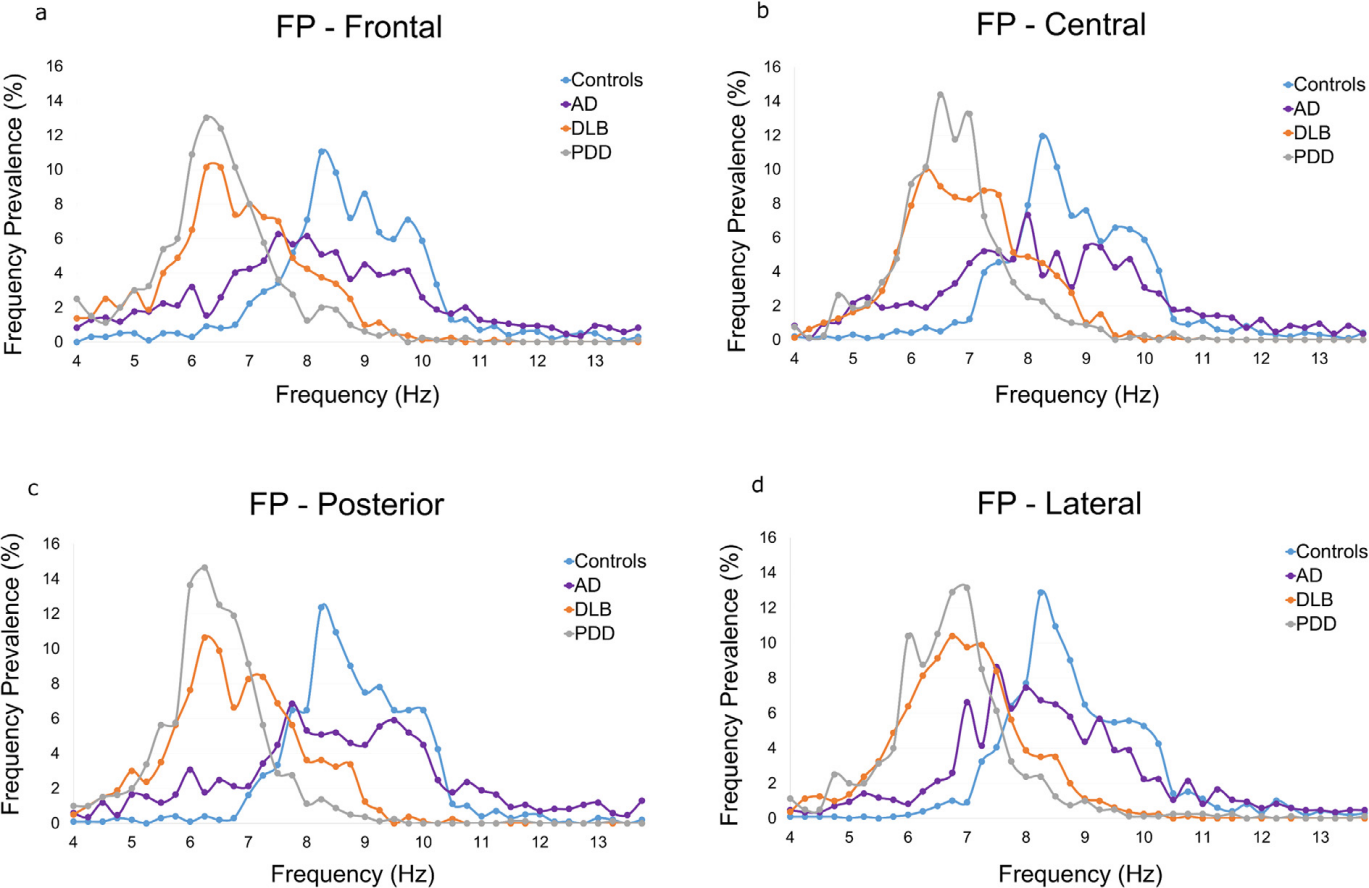
The mean frequency prevalence (FP; percentage distribution of the mean dominant frequency (DF) in each frequency point in the theta-alpha frequency range with 0.25 Hz resolution) for each of four diagnostic groups: healthy controls (N = 21), Alzheimer’s disease (AD; N = 18), dementia with Lewy bodies (DLB; N = 17) and Parkinson’s disease dementia (PDD; N = 17) patients, in the (a) frontal, (b) central, (c) posterior and (d) lateral regions.

For measures of frequency prevalence (FP; the percentage distribution of the theta-alpha DF in time in the slow-theta, fast-theta and alpha frequency ranges; Fig. 5, Table 3), the mean alpha FP was significantly higher in controls compared to all disease groups (*p < 0.01*), and in AD patients compared to DLB and PDD patients (*p < 0.01*), in all regions. In the fast-theta range the opposite pattern was observed, with controls exhibiting lower FP compared to AD patients frontally (*p < 0.01*), and to DLB and PDD patients in all regions (*p < 0.01*). Finally, in the slow-theta range controls had significantly lower FP than AD patients frontally (*p < 0.01*), centrally and posteriorly (*p < 0.05*), and to DLB and PDD patients in all regions (*p < 0.01*). AD patients also have significantly lower slow-theta FP than PDD patients frontally and centrally (*p < 0.05*).

### 3.3 Dominant frequency variability

**Fig. 6:**
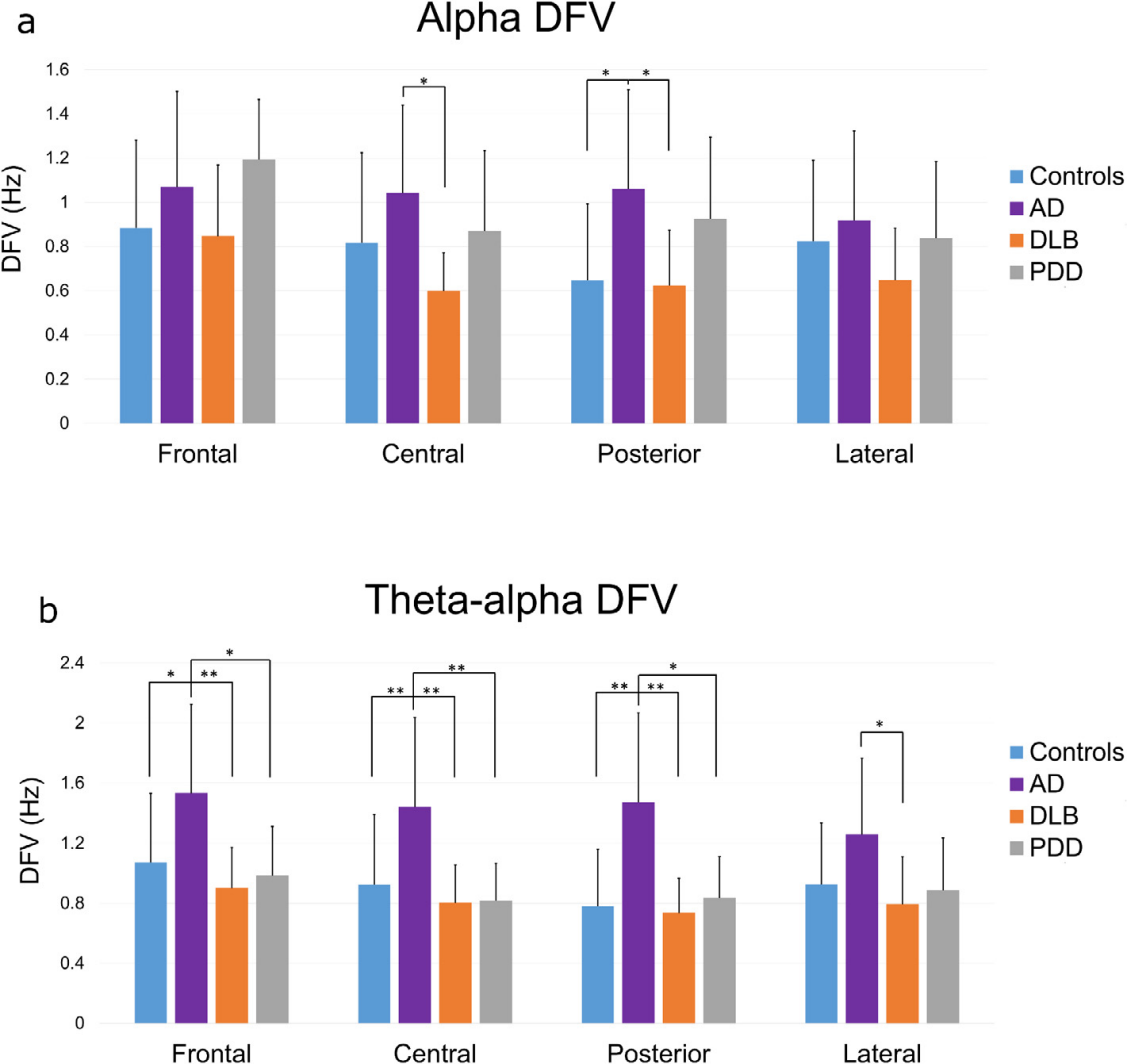
The mean dominant frequency variability (DFV) in the (a) alpha (8–13.5 Hz) and (b) theta-alpha (4–13.75 Hz) frequency ranges, for each of four diagnostic groups: healthy controls (N = 21), Alzheimer’s disease (AD; N = 18), dementia with Lewy bodies (DLB; N = 17) and Parkinson’s disease dementia (PDD; N = 17) patients, in the frontal, central, posterior and lateral regions. Error bards indicate the standard deviation (SD), ^**^p < 0.05, ^**^p < 0.01.

Comparisons of the DFV between groups for different frequency band and region combinations revealed a significant effect of diagnosis in the theta/alpha (*F (3, 69) = 2.77, p < 0.05* and alpha *(F (3, 69) = 6.29, p < 0.01)* ranges (Fig. 6), but not in the theta range. In the theta-alpha band, the AD group had a significantly higher DFV compared to the control, DLB and PDD groups in the frontal, central and posterior regions, and only to the DLB group laterally. In the alpha band, AD patients had significantly higher DFV compared to the DLB group centrally, and to DLB and controls posteriorly. To further validate this finding we have included a short analysis on the effect of each electrode on the DFV in AD and DLB patients (Supplementary Material 1).

### 3.4 Correlations

We assessed correlations between CFs as measured by CAF and DFV measures similarly to previous studies (Walker et al., 2000), and with QEEG measures of slowing for all the different diagnostic groups and each band and region. This analysis revealed that within the DLB group only, there was a strong correlation between the CAF score and the theta DFV in the central (*r = 0.789, p < 0.000*), posterior (*r = 0.652, p < 0.005*) and lateral regions (*r = 0.805, p < 0.000*). A positive, DLB specific correlation with CAF was also found with slow-theta FP in the frontal (*r = 0.679, p = 0.003*), central (*r = 0.747, p = 0.001*), posterior (*r = 0.792, p < 0.001*) and lateral (*r = 0.794, p = 0.001*) regions. A correlation between the CAF and MMSE score was only found in the PDD group (*r = −0.671, p < 0.05*), while no significant correlation was found for any variable and the LED, for any group and region.

### 3.5 Exploratory GEE and ROC curve analysis

**Fig. 7:**
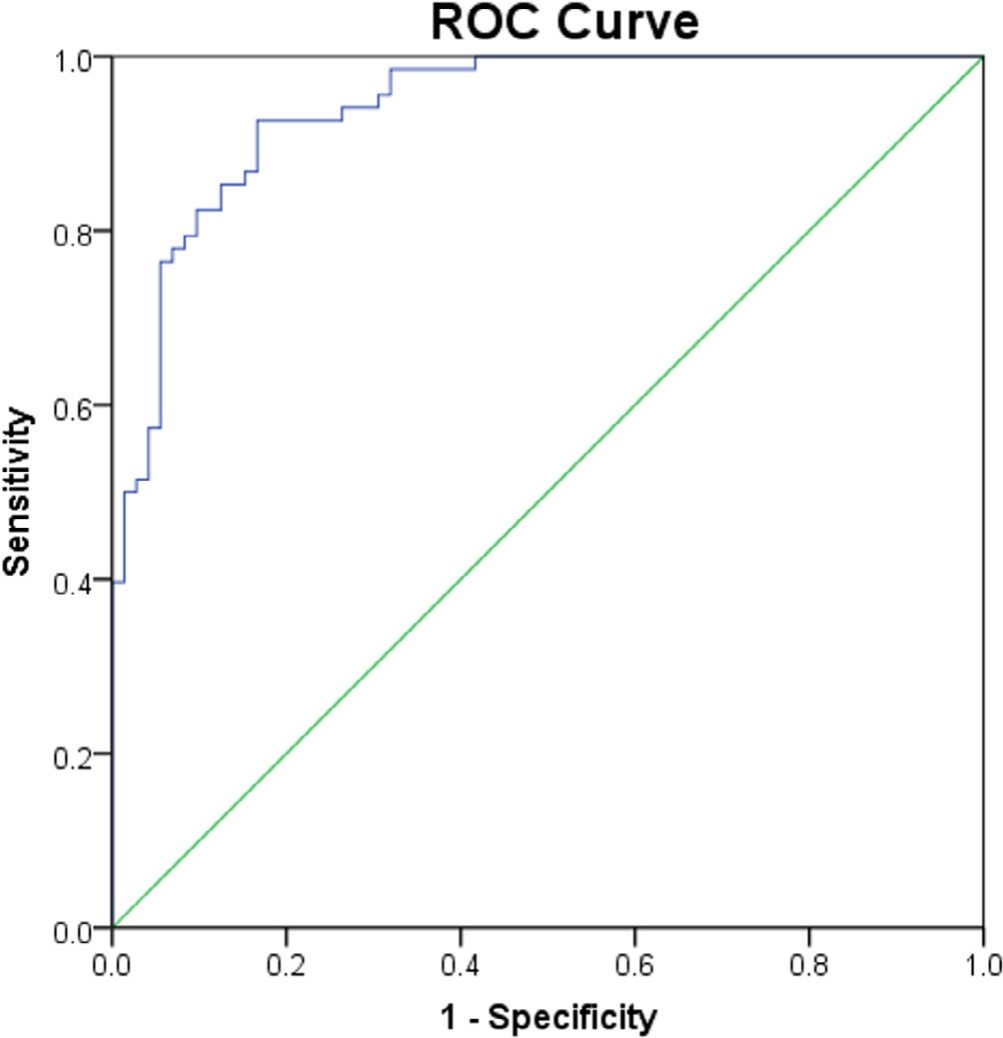
Receiver operating curves (ROC) for a model composed of fast-theta frequency prevalence (FP), theta power and theta-alpha dominant frequency variability (DFV), for differentiating between Alzheimer’s disease (AD; N = 18) and dementia with Lewy bodies (DLB; N = 17) with mild dementia.

GEE analysis was performed for the variables that were significantly different between the AD and DLB diagnostic groups (theta power, alpha power, theta-alpha DFV, alpha DFV, alpha DF and fast-theta FP). The alpha-theta DF and alpha FP were rejected from this analysis as they introduced marked multicollinearity. The QEEG variables that best predicted diagnosis were the theta power (%) (Wald chi-square = 15.74, df = 1, p < 0.01), the fast-theta FP (Wald chi-square = 8.1, df = 1, p < 0.01) and the theta-alpha SD (Wald chi-square = 7.549, df = 1, p < 0.01). ROC analysis (Fig. 7) yielded AUC = 94% (90.4–97.9%), sensitivity = 92.26% (CI = 80.4–100%) and specificity = 83.3% (CI = 73.6–93%). Since no significant differences were found between the PDD and DLB groups for any of the QEEG variables in the variance analyses, all the QEEG variables were included in the GEE analysis. This analysis deviated from the analysis protocol and is therefore included in the supplementary material (Supplementary Material 2), without drawing further conclusions.

## 4 Discussion

Our analysis has revealed several novel findings, including greater theta-alpha DFV in AD patients compared to controls, DLB and PDD patients. Moreover, we did not identify any differences in the DFV between the DLB group compared to controls, as was previously reported (Bonanni et al., 2016, 2008; Walker et al., 2000). However, we found a significant, DLB specific positive correlation between the CAF score and the theta DFV, and the CAF score and slow-theta FP. Our findings confirm the widely reported shift of EEG power and dominant rhythm – from the alpha towards the theta frequency range in the DLB and PDD groups compared to healthy controls and AD patients (Briel et al., 1999, Barber et al., 2000, Bonanni et al., 2008). A subtler slowing of the EEG was also observed in AD patients compared to controls. Finally, a preliminary analysis investigating the possible diagnostic value of QEEG variables showed that the three QEEG variables describing the extent of EEG slowing and DFV (theta power, fast-theta FP and theta-alpha DFV) could predict a DLB versus an AD diagnosis with high sensitivity and specificity.

A more marked EEG slowing in DLB/PDD groups compared to healthy controls and AD patients has been extensively reported in the literature, mostly in posterior derivations (Briel et al., 1999, Barber et al., 2000, Roks et al., 2008). In our analysis we looked within four different cortical regions compared to three regions previously reported (Bonanni et al., 2016, 2008), and analysed three measures of EEG spectral distribution, the FP, DF and power, all of which indicated a greater EEG slowing in DLB/PDD patients compared to AD patients and controls.

In AD patients, EEG slowing of a lesser extent was observed, that was evident by a shift of FP from the alpha to the fast-theta and slow-theta ranges compared to healthy controls. This finding indicates that a higher percentage of measurements of the theta-alpha DF in time fell in the theta-band rather than in the alpha-band in AD patients compared to controls. This altered DF distribution towards lower frequencies in AD was “masked” with the calculation of the mean theta-alpha DF, as this measure does not account for variability. The DF in the AD group is highly variable and can take values towards the higher edge of the alpha band thus influencing the mean DF. This is evident by the significantly greater theta-alpha DFV and the trends for greater alpha DFV and alpha DF in the AD group.

A cholinergic deficit may partly account for the EEG slowing in LBDs and AD, as the administration of AChEIs can reverse the EEG slowing in both diseases (Adler et al., 2004, Babiloni et al., 2013, Bosboom et al., 2009). However, the loss of cholinergic neurons projecting to the cortex is greater and has a faster progression in DLB and PDD compared to AD (Lippa et al., 1999) where the cholinergic deficit is not yet severe at mild stages of the disease (Bohnen and Albin, 2011). Pathological protein-related synaptic dysfunction that occurs before neuronal degeneration has also been associated with cognitive decline in AD and is thought to be even greater in DLB (Schulz-Schaeffer, 2010, Selkoe, 2002). Thus, a more advanced cholinergic deficit and synaptic dysfunction in the LBD groups could account for the greater extent of EEG slowing observed compared to the AD group, particularly given the relatively early disease stage/cognitive impairment that our participants exhibited.

Our analysis also revealed novel findings regarding temporal variability in the dominant rhythm as measured by DFV. Previous studies have shown a significant DFV increase in DLB patients compared to healthy controls, that correlated with CFs measured by CAF (Bonanni et al., 2008, Walker et al., 2000). Although we did not find an increase in the DFV of DLB patients compared to controls, we did find a positive correlation between theta DFV and the CAF score within the DLB group (Bonanni et al., 2015). This correlation was only significant in the theta frequency range, likely due to the shift of the DF towards these frequencies. A positive correlation was also found between slow-theta FP and the CAF score in DLB patients. Both these correlations were only seen in the DLB group and not in the PDD or AD groups.

Given the neuropathological similarities between PDD and DLB and the absence of other QEEG differences between these groups, the lack of a correlation between CAF and our QEEG measures in PDD was unexpected. Previous studies have reported that PDD patients with high CF scores show an EEG-slowing (Bonanni et al., 2008) and have more DLB-like symptoms such as visual hallucinations, while patients with lower CF scores resemble PD (Varanese et al., 2010). This PDD heterogeneity may have affected our capacity to identify a correlation between the EEG measures and CAF score in this group. Moreover, DLB patients with parkinsonism have more impaired reaction times and vigilance measures that relate to CFs, compared to patients without motor symptoms, implying a connection between CFs and dopaminergic impairment (Ballard et al., 2002). Since PDD is characterised by greater dopaminergic impairment than DLB, this additional pathology could have a more dominant aetiological role in the CFs seen in PDD as compared to DLB and thus be less contingent on factors (e.g. cholinergic tone) which might drive a QEEG change that associates with CFs. Furthermore, fluctuations are likely to have at least two dimensions (arousal and attention; Bliwise et al., 2012) which are not discriminated by the CAF scale but which may be differentially expressed in our DLB and PDD groups given arousal/sleepiness is strongly influenced by dopaminergic medications. Another factor may be the amyloid burden as this is significantly greater in DLB compared to PDD (Donaghy et al., 2015) and the cortical amyloid-β deposition relates more to dementia severity, visual hallucinations and delusion in DLB than PDD (McKeith et al., 2004). DLB is also characterised by a greater amyloid load in the putamen (Hepp et al., 2016), which is involved in attentional networks and in DLB has altered functional connectivity that correlates with CAF (Peraza et al., 2014). Improved quantification scales of fluctuations may help unpick these challenges.

Previous studies have also shown that DLB patients had a significantly higher DFV compared to AD patients, which did not differ significantly from controls, and that a higher DFV was an accurate indicator of DLB versus AD diagnosis (Bonanni et al., 2008). A QEEG analysis on the same patient cohorts as in this study, but with less spatial detail, also suggested a greater theta-alpha DFV in AD patients compared to the DLB/PDD groups, posteriorly (Peraza et al., 2018). Here, we found that AD patients had a significantly higher theta-alpha DFV compared to the other groups in most regions while DLB patients were not significantly different than PPD patients or controls. In the current study looking within smaller frequency bands in the theta-alpha range we also identified a greater alpha DFV in AD patients compared to controls and DLB patients posteriorly, and to DLB patients alone centrally. These findings could be part of the pathology or alternatively, the result of a compensation mechanism that may occur at early stages of AD. At rest, early stage AD patients may have increased activity and functional connectivity in resting state networks which correlate with a lower MMSE score (Peraza et al., 2016). However, at more advanced stages activity and connectivity decrease to levels lower than those seen in controls (Agosta et al., 2012). Therefore, increases in DFV may be associated with a compensation mechanism in early stage AD.

A number of other factors may account for the discrepancies between our findings and those of previous studies. The lack of a greater DFV in DLB patients compared to controls may be attributed to the fact that the majority of our DLB patients were on AChEIs, although we would argue that this adds to the clinical relevance of our findings, particularly from a diagnostic perspective; it is likely that any use of the EEG will be when patients are beginning or have already been initiated on treatment. In DLB patients, CFs have been shown to correlate with cholinergic imbalances in networks involved in the resting state (Delli Pizzi et al., 2015). AChEIs restore this imbalance and improve both the cognitive symptoms of DLB and the electrophysiological markers, including the EEG spectrum and connectivity (Onofrj et al., 2003). That said, it is important to acknowledge that more AD (94.4%) than DLB (88.2%) patients were on AChEIs in our study groups and the former group showed greater DFV. However, as outlined above, cholinergic deficits are greater and occur earlier in DLB compared to AD (Tiraboschi et al., 2002), while the brainstem cholinergic innervations of the thalamus are relatively spared in AD (Mesulam, 2004) but not in DLB (Taylor et al., 2017). Hence, at the stage of mild dementia AChEIs could have a differential effect in DLB and AD. Although AChEIs may have normalized the DFV in DLB patients in relation to healthy individuals, the CAF/DFV correlation was still maintained within the DLB group. In previous studies, none (Walker et al., 2000), or only a small proportion (Bonanni et al., 2008) of the patients were on AChEIs. Differences in the participant cohorts, as well as methodological differences in the analysis of the recordings must also be considered. Specifically, we used a different pre-processing and spatial analysis approach, as well as a different way to estimate DFV; here DFV was defined as the standard deviation from the mean DF across epochs, in an epoch-by-epoch basis, while in Bonanni et al., 2008, Bonanni et al., 2016, DFV was defined using a visual rating of DF range on sequential EEG segments.

Finally, we proceeded with a preliminary analysis to investigate the capacity of the QEEG variables to correctly differentiate between AD and DLB patients with mild dementia. The theta power, fast-theta FP and theta-alpha DFV yielded accuracy of 94% (CI = 90.4–97.9%), sensitivity of 92.26% (CI = 80.4–100%) and specificity of 83.3% (73.6–93%). The high predictive accuracy of this model is in-line with previous classifications using QEEG variables, although different EEG pre-processing and analysis methods were used (Andersson et al., 2008, Garn et al., 2017).

A few issues relating to this study need to be considered and an important next step would be the confirmation of our findings in independent prospective cohorts, especially regarding the ROC analysis. We excluded the delta frequencies and hence, we might have missed changes in the QEEG variables within that range. In addition, the recordings were not always continuous as we focused on discarding as much of the noise as possible and preferred to occasionally reject epochs, across all channels. Moreover, the patients did not undergo post-mortem immunohistological examination and thus we did not account for mixed AD-DLB pathology that has been shown to relate to greater cognitive impairment in DLB patients (Gomperts et al., 2012) and which may alter the QEEG pattern. However, our clinical diagnostic approaches were robust enough to enhance the specificity of our group selections. Evidence for this include DaT scans that were available for 9 of the DLB patients and were all positive, and a multi-modal MRI/EEG analysis on data from all the patients that were recruited in the same cohort as the patients included in this study, where AD and DLB patients were classified with 90% accuracy (Colloby et al., 2016).

## 5 Conclusions

Our findings confirm the well-established slowing of the EEG in the Lewy body dementia groups compared to healthy controls and AD patients. Although we did not find higher DFV in DLB patients compared to controls as expected, theta DFV and slow-theta FP were positively correlated with CFs as measured by CAF. This DLB specific correlation suggests that a slower and more temporally variable DF specifically relates to the CFs seen in DLB, and could reveal differential mechanisms underlying CFs in dementia subtypes. Another novel finding was a significantly higher DFV in AD patients compared to the other groups. Exploratory analysis showed that QEEG measures could predict a DLB versus an AD diagnosis with high accuracy, sensitivity and specificity. In conclusion, this study supports the hypothesis that QEEG analysis can be a valuable tool for identifying CFs in DLB and for differential diagnosis between dementia subtypes, once replicated with low density EEG currently used in standard clinical practice after the feasibility and cost-effectiveness of these methodologies has been investigated.
