# Supplementary material for: Quantitative electroencephalography as a marker of cognitive fluctuations in dementia with Lewy bodies and an aid to differential diagnosis
Source: Clin Neurophysiol. 2018 Jun;129(6):1209–20. doi: 10.1016/j.clinph.2018.03.013 (PMC5954167; doi:10.1016/j.clinph.2018.03.013)
Supplement: Supplementary data 1 [file mmc1.docx]

**Supplementary Material 1**

In order to further validate our findings regarding the dominant frequency variability (DFV) and to demonstrate that the approach of averaging the quantitative EEG variables across electrodes generally represents the single electrode level, we investigated the effect of posterior electrodes on the theta-alpha DFV, in Alzheimer’s disease (AD; n=18) and dementia with Lewy body (DLB; n=17) patients. Repeated measures ANOVA was carried out where the 35 posterior electrodes were classified as the within-subjects variable, and diagnosis as the between-subjects variable. The test of within-subjects effect with a Huyhn-Feldt correction due to violation of the assumption of sphericity, showed a non-significant effect of electrode (*F (10.927) = 2.469, p = 0.006*) when corrected for multiple comparisons (α = 0.001). The between-subjects variable (diagnosis) was highly significant (*F (1) = 21.769, p < 0.0001*). This finding agrees with our main statistical analysis using the DFV averaged across all posterior electrodes (Now Figure 6). The lack of significance for the effect of electrode and the clear effect of diagnosis demonstrates that using the average of this quantitative EEG variable across regional electrodes does not mask or favor any meaningful effects. The DFV was chosen for this analysis as it is a product of the mean DF and is used to calculate the frequency prevalence (FP). Hence we can extrapolate that the average for these QEEGs across electrodes is also representative of the data at the electrode level.


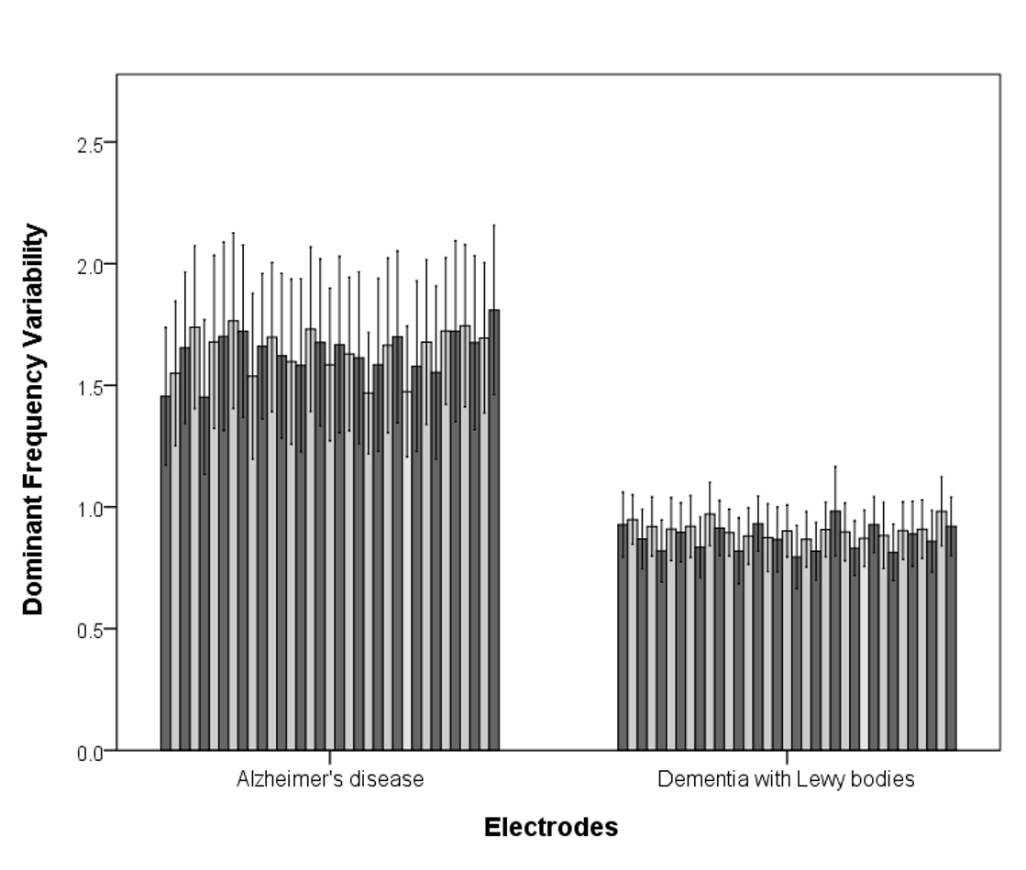


**Supplementary Figure.** Bar-chart representation of the theta-alpha (4 – 13.75 Hz) dominant frequency variability (DFV) calculated for each posterior electrode (n = 35), for the Alzheimer’s disease (n = 18) and dementia with Lewy bodies (n = 17) diagnostic groups. The error bars show the variability in DFV between subjects with a 95% confidence interval. The electrode order represented by the bars is corresponding between diagnostic groups from left-to-right.
