# Supplementary material for: Quantitative electroencephalography as a marker of cognitive fluctuations in dementia with Lewy bodies and an aid to differential diagnosis
Source: Clin Neurophysiol. 2018 Jun;129(6):1209–20. doi: 10.1016/j.clinph.2018.03.013 (PMC5954167; doi:10.1016/j.clinph.2018.03.013)
Supplement: Supplementary data 2 [file mmc2.docx]

**Supplementary Material 2**

No significant differences were found between the DLB and PDD patients and therefore, all QEEG variables were used for this analysis (theta power, alpha power, alpha DF, theta DF, theta-alpha DF, slow-theta FP, fast-theta FP, alpha FP, theta DFV, alpha DFV, theta-alpha DFV). The theta-alpha DFV, theta DF, slow-theta FP and alpha FP were removed from the analysis due to high multicollinearity (VIF > 5). The remaining variables underwent GEE analysis and the best predictors of diagnosis were the fast-theta FP (Wald chi-square = 7.551 df = 1, p < 0.01), theta-alpha DF (Wald chi-square = 6.312 df = 1, p < 0.05), alpha DF (Wald chi-square = 6.094 df = 1, p < 0.05) and theta power (Wald chi-square = 4.383 df = 1, p < 0.05). ROC analysis showed that this model could predict and DLB versus a PDD diagnosis with 78% accuracy, 70.6% sensitivity and 66.2% specificity.
